# Dermatopathological Findings of an Erythema Multiforme‐Like Drug Eruption During Enfortumab Vedotin Plus Pembrolizumab Therapy for Metastatic Urothelial Carcinoma: A Case Report

**DOI:** 10.1002/ccr3.71950

**Published:** 2026-04-03

**Authors:** Ryunosuke Murofushi, Yoichiro Kato, Takashi Seo, Kazuhiro Iwasaki, Maki Goto, Nobuhiro Monma, Shigekatsu Maekawa, Wataru Obara

**Affiliations:** ^1^ Department of Urology Morioka Red Cross Hospital Morioka Japan; ^2^ Department of Urology Iwate Medical University Shiwa‐gun Japan; ^3^ Department of Dermatology Morioka Red Cross Hospital Morioka Japan; ^4^ Department of Pathology Morioka Red Cross Hospital Morioka Japan

**Keywords:** dermatopathological findings, enfortumab vedotin plus pembrolizumab, erythema multiforme–like eruption, metastatic urothelial carcinoma

## Abstract

The combination therapy of enfortumab vedotin and pembrolizumab (EV+Pem) is effective for metastatic urothelial carcinoma. However, the dermatopathological findings for treatment‐related adverse events, particularly skin disorders, often remain unclear. We report a 69‐year‐old male with metastatic renal pelvic cancer who developed a Common Terminology Criteria for Adverse Events (CTCAE) grade 3 erythema multiforme–like drug eruption during EV+Pem on day 8 of cycle 3. Dermatopathological findings by skin biopsy at onset and remission were evaluated with immunohistochemistry, demonstrating improvement in proliferative activity (mitotic figures/Ki‐67) in parallel with clinical improvement. Peripheral blood tests showed parallel changes in monocytes and eosinophils with the clinical course of the eruption. Both agents were temporarily discontinued and systemic corticosteroids were initiated, followed by tapering. EV was resumed with two‐step dose reduction, while pembrolizumab was resumed at the full dose. However, grade 2 rash recurred and was controlled with prednisolone escalation and subsequent tapering, allowing continued therapy with further EV dose reduction. The metastatic lesions have remained in partial response (PR). The evaluation of dermatopathological findings at onset and remission is important in managing skin disorders associated with the combination therapy of EV and pembrolizumab in patients with metastatic urothelial carcinoma.

## Key Clinical Message

Despite histopathologic improvement, erythema multiforme–like eruption can recur during enfortumab vedotin plus pembrolizumab. Basal‐layer mitoses and high Ki‐67 support enfortumab/monomethyl auristatin E (MMAE) involvement. In our case, cautiously monitored, stepwise enfortumab rechallenge was feasible, underscoring that pathology guides causality assessment but not safety alone.

## 1 Background

The adverse events associated with the combination therapy of enfortumab vedotin and pembrolizumab (EV+Pem) for unresectable urothelial carcinoma (UC) require careful attention [1]. Although several reports on skin disorders and their management during monotherapy with enfortumab vedotin (EV) or pembrolizumab (Pem) have been published [2, 3], reports on combination therapy remain limited. In our case, the patient developed a CTCAE grade 3 erythema multiforme–like drug eruption after initiating combination therapy with EV+Pem for metastatic urothelial carcinoma. We present dermatopathological and immunohistochemical findings, along with longitudinal changes in differential white blood cell counts, observed at the onset and resolution of the drug eruption.

## 2 Case Presentation

**FIGURE 1:**
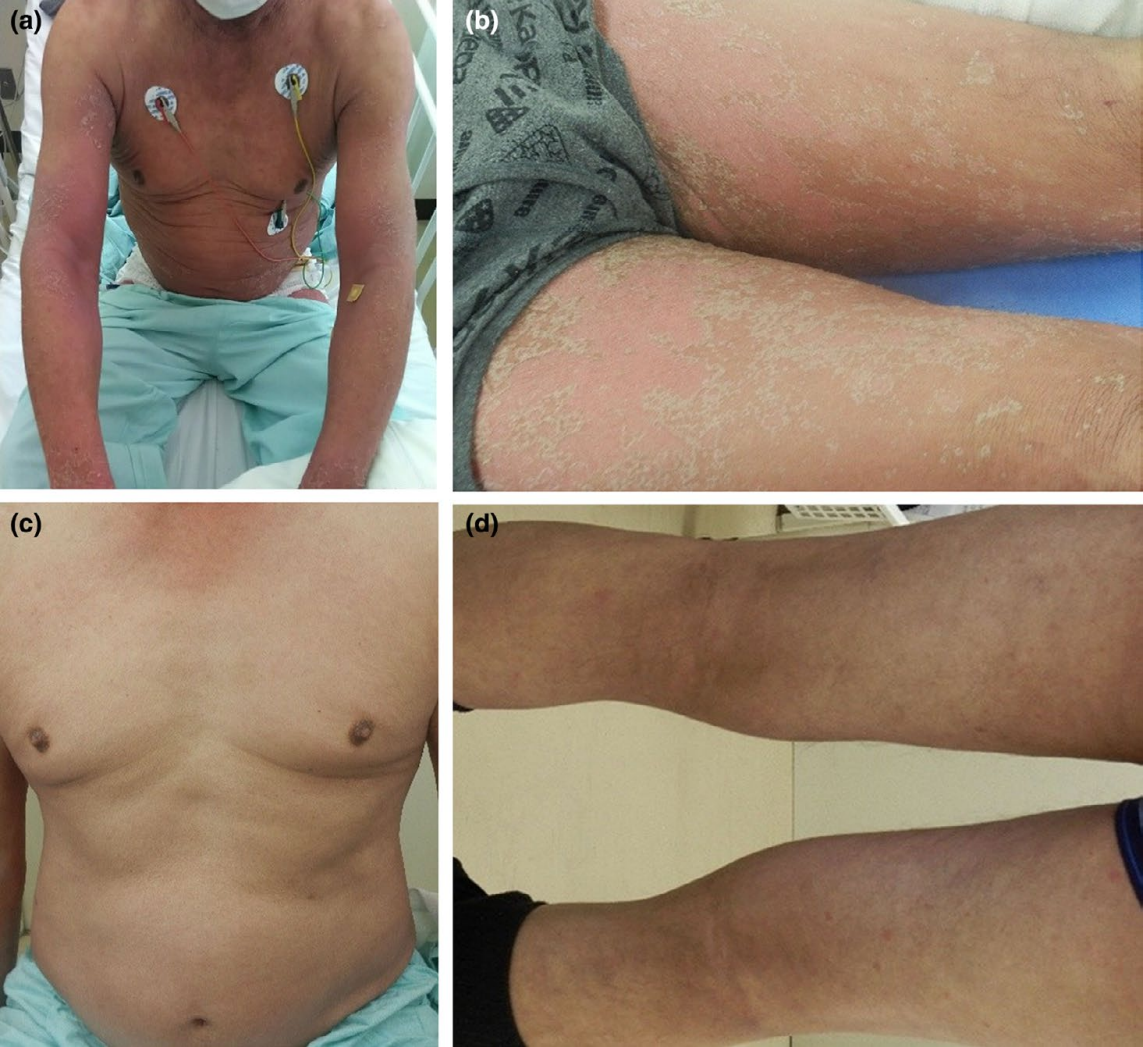
Clinical photographs of the skin eruption. (a, b) At onset (day 1), erythema multiforme‐like lesions on the trunk and extremities. (c, d) After improvement (day 49), the eruption markedly improved.

The patient was a 69‐year‐old man with a medical history of hypertension, chronic atrial fibrillation, diabetes mellitus, cerebral infarction, glaucoma, and chronic kidney disease (CKD stage G4), but no known history of allergies. Based on the diagnosis of right renal pelvis carcinoma, a laparoscopic right nephroureterectomy was performed. Pathological examination revealed invasive urothelial carcinoma, pT3N0M0. Three months postoperatively, CT and cystoscopy revealed multiple liver and lung metastases with intravesical recurrences. Combination therapy with EV (1.25 mg/kg) + Pem (200 mg) was initiated. On day 8 of the third treatment cycle, he developed a widespread grade 3 erythema with pruritus involving the trunk and extremities, accompanied by a fever of 38.2°C (Figure 1a,b). He was admitted immediately (day 1).

## 3 Dermatopathology Findings

**FIGURE 2:**
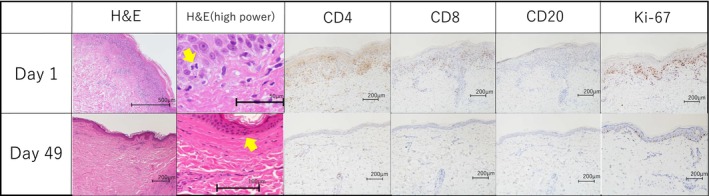
Pathological and immunohistochemical examination by skin biopsy. (Upper panel) Onset (day 1). H&E staining showed parakeratosis in the epidermis. In the high‐power H&E image, the arrow indicates a basal‐layer mitotic figure. Immunohistochemistry demonstrated a CD4‐predominant T‐cell infiltrate compared with CD8‐positive cells. Ki‐67 positive cells were observed in the basal layer of the epidermis. (Lower panel) Pre‐resumption (day 49). H&E staining showed no remarkable epidermal abnormalities, and mitotic figures were not observed. Immunohistochemistry for CD4, CD8 and CD20 showed no remarkable inflammatory cell infiltrates. Ki‐67‐positive cells were sparse in the basal layer compared with the onset biopsy. Scale bars: (Upper panel) 500 μm (H&E), 50 μm (high‐power H&E) and 200 μm (CD4, CD8, CD20, Ki‐67). (Lower panel) 100 μm (high‐power H&E) and 200 μm (H&E, CD4, CD8, CD20, Ki‐67).

A skin biopsy was performed on admission (day 1) at the recommendation of a dermatologist. Histopathological examination showed parakeratosis in the epidermis, epidermal necrosis, subepidermal blister formation, and inflammatory infiltration of the basal layer. Marked mitotic activity was also observed in basal keratinocytes (Figure 2). In the clinical context, these findings were consistent with an erythema multiforme (EM)‐like drug eruption. Immunohistochemical analysis revealed CD4‐predominant lymphocytic infiltration, with an approximate CD4/CD8 ratio of 2:1. CD20‐positive cells were sparse, and numerous Ki‐67–positive cells were observed in the basal epidermal layer.

A repeat skin biopsy was performed on day 49. Compared with the initial biopsy (day 1), the acute histopathological findings had markedly improved, and mitotic figures in the basal layer were no longer observed (Figure 2). Immunohistochemical analysis revealed minimal lymphocytic infiltration; the CD4/CD8 ratio remained unchanged at 2:1, CD20‐positive cells were extremely scarce, and Ki‐67‐positive cells had decreased to approximately one‐tenth of the level observed in the initial biopsy.

## 4 Management and Course

On admission, peripheral blood tests showed an elevated monocyte count of 680/μL, accounting for 14.9% of the total white blood cell count. CT revealed a marked reduction in liver and lung metastases. After admission, combination therapy with EV+Pem was discontinued. Intravenous prednisolone was initiated at 70 mg/day on admission and transitioned to oral prednisolone at 60 mg/day on day 5, followed by a taper. From day 1, an oral antihistamine (bepotastine besilate, 10 mg/day) and topical corticosteroids were initiated and continued throughout the course. Treatment interruption, rechallenge, and dose modification were guided by CTCAE‐based severity grading, the prescribing information for EV and pembrolizumab, and recommendations from pivotal trials and prior reports. The skin symptoms subsequently improved (Figure 1c,d). The dose of prednisolone was tapered gradually to 20 mg/day by day 30, and he was discharged on day 32. The peripheral blood test after discharge (day 49) showed improvement, with a monocyte count of 431/μL and a monocyte percentage of 6.0%, consistent with the improvement of the drug eruption. Based on clinical and pathological findings, the EM‐like drug eruption was judged to have improved. Prednisolone was continued after discharge and was tapered to 7.5 mg/day by day 56. On day 59, EV was resumed at 0.75 mg/kg in a two‐step reduction, while Pem was resumed at 200 mg. However, on day 66, edematous erythema primarily involving the trunk emerged. Based on the dermatologic features and overall clinical status, the eruption was assessed as grade 2, and prednisolone was increased to 20 mg/day. On day 8 (day 66) of the fourth cycle, EV was not administered. The fifth cycle (day 80) was initiated with a three‐step dose reduction to 0.5 mg/kg of EV and Pem was continued at the same dose. During rechallenge, he was evaluated at each scheduled infusion visit with symptom assessment and laboratory monitoring, and dermatology follow‐up was coordinated with these visits. Prednisolone is being tapered and is currently administered at 7 mg/day. The metastatic lesions have remained in partial response.

## 5 Discussion

Skin disorders are known adverse events associated with the combination therapy of EV+Pem. The incidence rate of grade 3 or higher maculopapular rash with EV+Pem combination therapy has been reported as 7.7% [1]. It is important to take adequate measures and management for skin disorders. In this case, grade 3 skin disorder, erythema with pruritus involving the trunk and extremities was observed during the third cycle of EV+Pem. Pathological findings by skin biopsy showed epidermal necrosis, subepidermal blister formation, and inflammatory infiltration of the basal layer. Based on clinical symptoms and pathological findings, erythema multiforme (EM)–like drug eruption was diagnosed. In this case, the skin symptoms improved following discontinuation of treatment and administration of steroids.

We investigated the causative drug of EM from several perspectives. At the onset of EM, skin biopsy findings revealed marked mitotic activity in the basal layer keratinocytes. Moreover, immunohistochemical analysis revealed a high number of Ki‐67–positive cells in the basal epidermal layer. As reported by Rosenberg et al., EV is an antibody–drug conjugate. Nectin‐4 is highly expressed in urothelial carcinoma [2]. The anti–Nectin‐4 antibody binds to Nectin‐4 antigen on the surface of Nectin‐4 expressing cells forming a complex. The complex is internalized within the cell and is trafficked to lysosomes. Monomethyl auristatin E (MMAE) is intracellularly released after lysosomal cleavage of the valine–citrulline linker, binds microtubules, halts tubulin polymerization, and induces G2/M arrest followed by apoptosis. Sport et al. emphasized the significance of starburst‐like mitotic figures and ring‐shaped mitotic changes as characteristic histopathological features of EV‐induced rash [4]. In our case, mitotic figures were prominent at the onset of the EM‐like drug eruption but disappeared following corticosteroid treatment. Similarly, Ki‐67 expression was markedly elevated during the acute phase and decreased to approximately one‐tenth of its initial level prior to treatment resumption. These findings suggest that M‐phase arrest caused by EV may have occurred during the active phase of the rash. Hirotsu et al. reported that mitotic figures in the basal layer of the epidermis are an important characteristic finding indicating the action of MMAE on EV [5].

We also examined the expression of immune cells by immunohistochemistry to investigate the potential for Pem‐induced EM. At the onset of EM, CD4‐positive lymphocytes were markedly elevated, with a CD4/CD8 ratio of 2:1. After EM improvement, the ratio remained the same, but CD4‐positive lymphocytes had decreased. CD20‐positive cells were sparsely present both before and after EM onset. Kadoi et al. reported a case of anal canal mucinous adenocarcinoma with multiple lymph‐node metastases in which pembrolizumab was initiated after systemic chemotherapy and repeated radiotherapy to cutaneous metastases, and immunohistochemical analyses were performed [6]. Their case was diagnosed as EM and showed CD4 predominance. However, compared with our case, key differences include the tumor type, the absence of a description of mitotic figures, the background of prior cutaneous irradiation, the timing of rash onset, the steroid dosing and time to response, and whether treatment was reinitiated. Considering the basal‐layer mitoses and the patient's clinical course, these findings suggest enfortumab vedotin as a possible cause.

**FIGURE 3:**
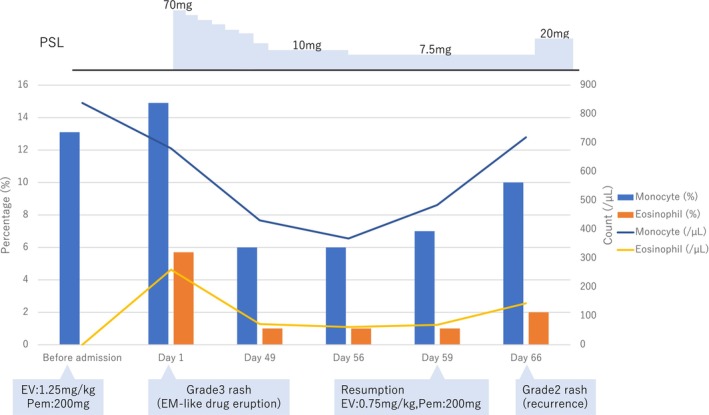
Changes in peripheral blood monocyte and eosinophil percentages and absolute counts in relation to the clinical course. Key clinical events are annotated.

In our case, monitoring of peripheral blood monocyte counts and percentages was conducted in parallel with skin findings and histopathological findings (Figure 3). Notably, a reproducible increase in monocyte counts and percentages was observed at the onset of grade 3 EM and at the recurrence of grade 2 rash after rechallenge. These findings suggest that monocyte dynamics may reflect systemic inflammation accompanying EV+Pem‐associated cutaneous toxicity; however, evidence is limited and warrants validation. Elevation of monocytes may serve as a useful marker for monitoring skin disorder associated with EV+Pem therapy.

In the setting of EV plus pembrolizumab, temporary treatment interruption and dose modification are often required to manage dermatologic adverse events while maintaining therapy. Brower et al. suggested that, in cases with rapid worsening or severe reactions, both agents should be temporarily interrupted and systemic corticosteroids should be initiated, with rechallenge considered after improvement to CTCAE grade ≤ 1 [7]. They also proposed that rechallenge should be considered when prednisolone has been tapered to a prednisone‐equivalent dose of ≤ 10 mg/day (in our case, prednisolone 7.5 mg/day). The prescribing information for EV likewise recommends temporary interruption for CTCAE grade 3 cutaneous reactions, with rechallenge including dose reduction after improvement, and consideration of discontinuation for severe cutaneous adverse reactions. Overall, the interruption/rechallenge strategy and dose modification in this case were largely consistent with CTCAE‐based severity grading and existing recommendations. In summary, if cutaneous toxicity rapidly worsens or reaches CTCAE grade ≥ 3, both EV and pembrolizumab should be temporarily discontinued. Rechallenge can be considered after improvement to CTCAE grade ≤ 1 and tapering corticosteroids to a prednisone‐equivalent dose of ≤ 10 mg/day, with EV resumed with dose reduction as appropriate. There is some controversy regarding the resumption of treatment after improvement of dermatologic adverse events. Among the 15 previously reported cases [8, 9, 10, 11, 12, 13, 14], EV+Pem was resumed in 6 cases (with EV dose reduction in 4 cases), Pem alone was resumed in 4 cases, and EV alone was resumed in 1 case. Both agents were discontinued in 3 cases. In the remaining 1 case, EV+Pem was discontinued and the patient subsequently transitioned to avelumab maintenance therapy after achieving complete response (CR). In total, including the present case, 7 out of 16 cases resumed EV+Pem, either at full dose or with dose reduction. However, in this case, despite a two‐step dose reduction and histopathological improvement, grade 2 skin lesions recurred upon re‐administration. This outcome underscores that histological improvement alone may not reliably predict the safety of treatment resumption. It highlights the need for cautious, individualized decision‐making when considering re‐initiation of EV+Pem therapy following dermatologic adverse events.

This report has several limitations, most notably its nature as a single case study. Although mitotic figures and Ki‐67 expression were confirmed during the onset of EM, the distribution of M‐phase cells was not specifically examined. As a result, it remains unclear whether the observed skin reaction was primarily driven by EV, by the lymphocytic proliferation associated with the inflammatory response to erythema multiforme, or by a combination of both.

## 6 Conclusion

The evaluation of dermatopathological findings at onset and remission is important in managing skin disorders associated with the combination therapy of EV and pembrolizumab in patients with metastatic urothelial carcinoma.

## Author Contributions

**Ryunosuke Murofushi:** conceptualization, data curation, investigation, project administration, resources, validation, visualization, writing – original draft. **Yoichiro Kato:** data curation, investigation, methodology, supervision, validation, visualization, writing – review and editing. **Takashi Seo:** investigation, validation, writing – review and editing. **Kazuhiro Iwasaki:** investigation, validation, writing – review and editing. **Maki Goto:** investigation, validation, writing – review and editing. **Nobuhiro Monma:** investigation, methodology, validation, writing – review and editing. **Shigekatsu Maekawa:** supervision, writing – review and editing. **Wataru Obara:** supervision, writing – review and editing.

## Funding

The authors have nothing to report.

## Consent

Written informed consent for publication was obtained from the patient using the Clinical Case Reports patient consent form.

## Conflicts of Interest

The authors declare no conflicts of interest.

## Data Availability

Data sharing is not applicable to this article as no new data were created or analyzed.
